# Wheel and Deal in the Mitochondrial Inner Membranes: The Tale of Cytochrome *c* and Cardiolipin

**DOI:** 10.1155/2020/6813405

**Published:** 2020-04-17

**Authors:** Antonio Díaz-Quintana, Gonzalo Pérez-Mejías, Alejandra Guerra-Castellano, Miguel A. De la Rosa, Irene Díaz-Moreno

**Affiliations:** Instituto de Investigaciones Químicas (IIQ), Centro de Investigaciones Científicas Isla de la Cartuja (cicCartuja), Universidad de Sevilla – Consejo Superior de Investigaciones Científicas (CSIC), Avda. Américo Vespucio 49, Seville 41092, Spain

## Abstract

Cardiolipin oxidation and degradation by different factors under severe cell stress serve as a trigger for genetically encoded cell death programs. In this context, the interplay between cardiolipin and another mitochondrial factor—cytochrome *c*—is a key process in the early stages of apoptosis, and it is a matter of intense research. Cytochrome *c* interacts with lipid membranes by electrostatic interactions, hydrogen bonds, and hydrophobic effects. Experimental conditions (including pH, lipid composition, and post-translational modifications) determine which specific amino acid residues are involved in the interaction and influence the heme iron coordination state. In fact, up to four binding sites (A, C, N, and L), driven by different interactions, have been reported. Nevertheless, key aspects of the mechanism for cardiolipin oxidation by the hemeprotein are well established. First, cytochrome *c* acts as a pseudoperoxidase, a process orchestrated by tyrosine residues which are crucial for peroxygenase activity and sensitivity towards oxidation caused by protein self-degradation. Second, flexibility of two weakest folding units of the hemeprotein correlates with its peroxidase activity and the stability of the iron coordination sphere. Third, the diversity of the mode of interaction parallels a broad diversity in the specific reaction pathway. Thus, current knowledge has already enabled the design of novel drugs designed to successfully inhibit cardiolipin oxidation.

## 1. Introduction

Mitochondria—the so-called powerhouses of the cell—are responsible for a broad assortment of metabolic processes. Their key role in cells is reflected by the cornucopia of proteins involved in its function. In total, more than 1150 genes related to organelle function are recorded in the human MitoCarta. Furthermore, 1 in every 5000 people are effected by a mitochondrial disorder [1].

Mitochondria play a significant role in cell homeostasis by helping to modulate cell signaling pathways. On one hand, the activity of the electron transport chain (ETC) is related to the release of reactive oxygen species (ROS) [2] which are strong modifiers of cell constituents such as proteins, nucleic acids, and lipids. Dysregulation of ROS can lead to oxidative stress which in turn can initiate cell death programs [3, 4], in which lipid peroxidation and their products play a key role [5].

Cardiolipin (CL) oxidation by cytochrome *c* (C*c*) at the onset of apoptosis is a decisive step [6]. During homeostasis, the soluble cationic hemeprotein is located in the mitochondrial intermembrane space, shuttling electrons between complexes III (CIII) and IV (CIV) in the ETC. Indeed, C*c* is a key Janus catalyst of CL signaling rather than a passive messenger. Its ability to oxidize superoxide anions (O_2_^-•^) to molecular O_2_ along with its peroxidase activity in solution reduce the damage caused by oxidative stress [7–12]. However, rearrangement of the mitochondrial membrane triggered by t-Bid upon severe stress makes CL available to bind C*c* [13] on the outer leaflet of the IMM. Thus, acyl chains of CL are oxidized due to the oxygenase activity of the hemeprotein [14]. In fact, oxygenase activity of C*c* rises substantially in C*c*-CL complexes [15]. Subsequent CL oxidation favors the release of C*c* into the cytosol where it triggers apoptosis [16–18]. Furthermore, an array of products from C*c*-mediated CL oxidation—e.g., hydroxy-, oxo-, and peroxipolyunsaturated fatty acids—act as cell fate decision signals [19].

Although major features of cell death signaling pathways converging on CL metabolism have been thoroughly characterized, understanding the intimate mechanism of CL oxidation by C*c* remains challenging. Both CL-containing membranes and C*c* display complex behaviors that depend on different factors, including experimental conditions and post-translational modifications (PTM) of the protein.

This review article aims to provide the readers with an overview of the interaction between C*c* and CL and how it affects the peroxidase and oxygenase activities of the hemeprotein. Particular emphasis will be made on the conformational plasticity of C*c*, which enables its Janus functionality. In addition, we will discuss free oxidation of CL, regulation of C*c* activity, and its relationship with a diverse range of human diseases and recent strategies to combat them.

## 2. Cardiolipin: Properties and Role in the Mitochondrial Membranes

Cardiolipins (1,3-bis(*sn*-3′-phosphatidyl)-*sn*-glycerol) are a group of anionic phospholipids found in the plasma membrane of various bacteria and the inner mitochondrial membrane of eukaryotic cells [20]. These lipids contain two 1,2-diacyl-*sn*-glycero-3-phos-phoryl moieties bridged by a glycerol molecule. The two phosphatidyl groups are stereochemically nonequivalent, being respectively in *pro-R* and *pro-S* positions with respect to carbon 2 in the bridge [21]. The presence of 4 acylation sites—a fifth one at the central carbon of the glycerol bridge is also possible—would be consistent with a diverse range of CL species according to the distinct acyl chains available in a given organism. In humans, for instance, we would expect 14^4^ CL derivatives. This contrasts with the rather lower diversity of CL compounds found in each organism [22].

Despite the presence of two phosphate groups in CL, it is thought that the single anionic species predominates. In this species, one proton is shared through a bicyclic resonance structure involving the central hydroxyl group [23]. In membranes, the glycerol hydroxyl forms intra- and interlipid hydrogen bonds with oxygen atoms from phosphate, not with carbonyl groups [24]. Early measurements of ionization constants yielded a first p*K*_A_ value of 2.8 and a second one in the range between 7.5 and 9.5. A recent fourier transform infrared spectroscopy (FT-IR) analysis on liposomes also suggests two ionization steps with p*K*_A_ values 4.7 and 7.9 [25, 26]. Density functional theory level computations indicate a wide gap between the two p*K*_A_ values [27]. Other results indicate the opposite: both behave as strong dibasic acids with p*K*_A_ values within the pH range 2-3 in solution [28] and membrane preparations [29, 30]. According to this data, membrane-embedded CL carries two negative charges at physiological pH values.

The behavior of CL-containing membranes is complex and strongly dependent on the composition [31] and experimental/simulation conditions [32]. For instance, the selected CL protonation state in deterministic simulations can influence results. Thermodynamic analyses with lipid mono- and bilayers indicate a decrease in the area compressibility modulus [33]. According to molecular dynamics (MD) simulations, their thickness—measured as interphosphate distances—decreases with CL content, as the electron density does [24, 34]. Furthermore, small-angle X-ray scattering (SAXS) and neutron scattering (SANS) have confirmed that CL-containing bilayers have a lower thickness. This may reflect the smaller head-group volume per phosphate. However, these membranes show larger distances between electron density maxima and a thicker hydrocarbon moiety [24]. Comparison of different MD trajectories of bilayers with PDB files suggests conformational selection takes place when CLs bind to membrane proteins [34]. The negative charge of CL and its four acyl groups strongly affect the phase preference of the lipid, which varies from lamellar (L*α*) to inverted hexagonal (H_II_) depending on pH [30, 32].

**Figure 1:**
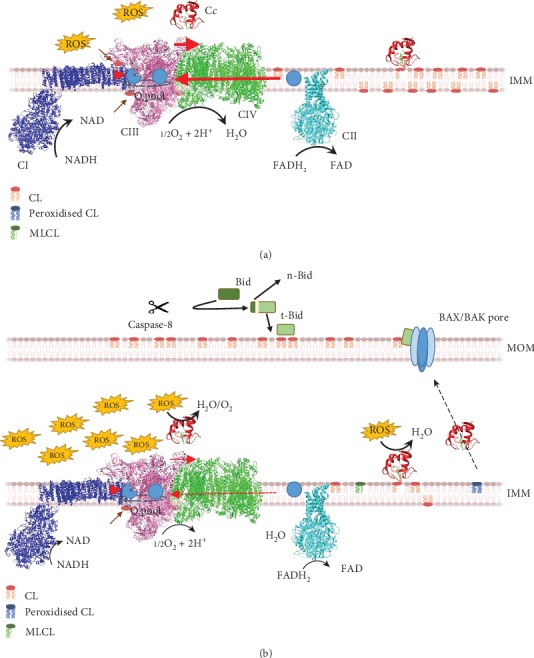
Role of cardiolipin in cell homeostasis and apoptosis. (a) Under homeostatic conditions, cardiolipin (CL) facilitates the assembly of respiratory supercomplexes (brown arrows) and maintains a population of C*c* bound to the inner mitochondrial membrane (IMM). The efficiency of electron transfer is high (thick red arrow). (b) Under apoptotic stimuli, procaspase-8 is recruited to CL-enriched microdomains in the outer mitochondrial membrane (OMM). The activation of caspase-8 involves cleavage of the BID proapoptotic factor into two domains, namely, the N-terminal (n-Bid) and C-terminal fragments (t-Bid). Dissociation of these two fragments is required for the interaction of t-Bid with CL. Then, t-Bid promotes the formation of mitochondrial pores by assembling BAX–BAK oligomers. At the same time, ROS production increases and C*c* acts as a ROS scavenger and pseudoperoxidase. C*c* peroxidase activity results in oxidation of CL acyl chains, to which the hemeprotein is anchored, freeing C*c* from the IMM, facilitating its subsequent release into the cytosol upon OMM permeabilization. The efficiency of electron transfer is low (dashed red arrow). In addition, CL can be degraded in part, losing one of its acyl chains, giving rise to monolysocardiolipin (MLCL).

CL is essential for the functionality of mitochondrial membranes and processes taking place therein—e.g., protein import and electron transport [6]. It represents between 5% and 20% of the total lipid content of the inner mitochondrial membrane (IMM) and is more abundant in the internal leaflet [35, 36] (Figure 1(a)). CL acts on membrane components of the ETC, aiding the assembly of the so-called respiratory supercomplexes [37, 38]. Supercomplexes modulate the performance of mitochondrial electron transport and oxidative phosphorylation [39]. Reportedly, CL is able to trap protons [40, 41], and it has been hypothesized to be important in the mechanism of CIII and IV acting as a proton exchanger [42–44]. The absence and/or modification of CL cause the development of several pathologies such as Barth's syndrome [37, 45–48]. Indeed, alteration of the IMM due to a decrease in the content of CL disrupts the ETC, increasing the generation of ROS [49] (Figure 1(b)). Remarkably, CL can be oxidized directly by ROS such as hydroxyl radicals and singlet oxygen, acting the products as proapoptotic signals [50].

CL is a mitochondrial stress-signaling factor in mitophagy and both the intrinsic and extrinsic apoptotic pathways [6, 51]. Under stress conditions (e.g., treatment with rotenone, staurosporine or cyclosporine A, and autophagic or apoptotic stimuli), CL molecules flip from the IMM to the outer mitochondrial membrane (OMM) [52–54] (Figure 1(b)). When eliciting the extrinsic apoptotic pathway in lymphoblastoid cells (type II cells) derived from Barth's syndrome patients and tafazzin knock-down HeLa cells, CL microdomains on the OMM recruit procaspase-8 to promote its activation [55, 56]. When caspase-8 becomes active, it cleaves the proapoptotic factor Bid, a BH3-only member of the Bcl-2 family [56]. The active C-terminal fragment of the Bid (t-Bid) targets CL or its degradation product monolyso-CL (MLCL) in mitochondria [57–60] and promotes OMM permeabilization [61]. During this process, the peroxidase activity of C*c* results in the oxidation of CL (to which it is anchored) facilitating the release of C*c* from the IMM and subsequent massive release into the cytosol at the onset of apoptosis [18, 62]. Extramitochondrial C*c* molecules interact with a variety of targets in the cytosol and nucleus, leading to a point of no return in the programmed cell death regulation [63–77].

## 3. Cytochrome *c* Binds Cardiolipin: A Tale of Grooves, Cavities, and Melting

C*c* belongs to the class I single-heme cytochrome *c* family, displaying the four typical *α*-helices conserved in the whole domain family [78]. In addition, C*c* displays three *Ω*-loops, two of them providing axial ligands for the heme iron. His18 at the proximal side of the heme provides the imidazole ligand conserved among the class I family. At physiological pH values, Met80 thioether acts as a distal ligand. The heme porphyrin ring is covalently bound to the protein backbone by thioether bonds between the vinyl groups of the porphyrin and conserved cysteine residues in the CXXCH motif. For human C*c*, conserved cysteine residues are Cys14 and Cys17. According to hydrogen exchange (HX) experiments, the apparently simple structure hides five folding units (called foldons) with different stabilities [79]. The most stable one (I) comprises the N- and C-terminal *α*-helices. Foldon II comprises *Ω*_I_ (from Thr19 to Phe36) and *α*-helix 3, which comes before *Ω*_III_. Foldon III (a.k.a. neck) comprises two short amino acid stretches with an extended conformation flanking the *Ω*_II_-loop. Notably, the latter faces heme propionates and is the least stable foldon (V), followed by the *Ω*_III_ region (IV) containing Met80.

The low stability of the loop containing Met80, comprising the sixth iron ligand, has a crucial role in C*c* physiology. Recent ultrafast X-ray spectroscopy analyses have highlighted the weakness of the Met80-S_*δ*_-Fe^+2^ bond and the lack of stability (4 kJ mol^−1^) provided by the protein matrix, most likely via hydrogen bonding [80].

At physiological pH, C*c* has a net charge of +8 from its unevenly distributed ionizable groups [81, 82]. This favors interactions with negatively charged molecules, such as the polar head of phospholipids, including CL. This interaction was first analyzed by Kimelberg and Lee, using lecithin-CL vesicles [83]. Their analysis together with early HX measurements by solid-state nuclear magnetic resonance (ssNMR) indicated that C*c* preferentially binds with CL [84]. They also suggested that during this interaction, CL destabilizes or unfolds the hemeprotein. Further relaxation time measurements by ^31^P NMR showed that C*c* impacts CL dynamics [85]. Surface plasmon resonance and electrochemical experiments on planar lipid bilayers allowed Salamon and Tollin to propose a two-step mechanism [86–88]. According to their proposal, C*c* first binds to membranes through electrostatic interactions and, subsequently, through hydrophobic interactions to promote changes in both the structure of C*c* and the membrane. Then, electron paramagnetic resonance (EPR) and magnetic circular dichroism (MCD) analyses showed that C*c* undergoes structural changes which affect Fe coordination and result in the appearance of a radical at high liposome concentrations [89]. Hence, mixing C*c* with lipids may yield several species, found in recent fluorescence anisotropy analyses [90].

**Figure 2:**
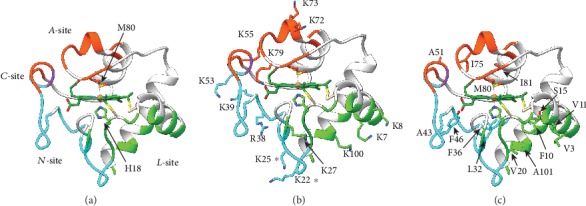
Cardiolipin-binding sites in cytochrome *c*. (a) Ribbon representation of oxidized human C*c* (PDB 2N9J) [92]. CL-binding sites are highlighted in orange (*A*-site or distal productive), green (*L*-site or proximal productive), purple (*C*-site), and cyan (*N*-site or proximal unproductive). The heme axial ligands H18 and M80 are highlighted as well. (b) Side chain representation of the positively charged C*c* residues involved in the formation of the C*c*-CL complex. Residues marked with an asterisk are reported to constitute the *L*- and *N*-sites. (c) Side chain representation of hydrophobic C*c* residues, which ensure the tight interaction between C*c* and CL acyl chains.

Apparently at odds with this proposed model, paramagnetic-quenching EPR experiments on horse heart C*c*, spin-labelled at different lysine positions, indicated that the hemeprotein can weakly interact with 1,2-dioleoyl-*sn*-glycero-3-phosphoglycerol (DOPG) bilayers [91]. This study highlighted three lysine residues at *Ω*_III_ (K72, K86, and K87, a.k.a *A*-site; Figure 2(a)) adjacent to the DOPG membrane. Further fluorescence studies using vesicles containing fluorescent lipid probes identified a secondary CL-binding site at low pH values [93]. Contrary to the *A*-site, CL association at this novel region (a.k.a. *C*-site; Figure 2(a)) is unaffected by ionic strength or the presence of ATP. Data suggested CL binds to this site via hydrogen bonds at N52, and a single acyl chain of the phospholipid inserts into a nearby hydrophobic pocket while the others remain in the bilayer [94]. This proposed interaction mechanism is known as the extended lipid anchorage model and is supported by studies on the ability of natural and engineered phospholipids to quench the fluorescence of Zn-substituted C*c* [95, 96]. Consistently, a N52I mutation heavily impacts the kinetics of the interaction between C*c* and CL in CL-containing liposomes [97].

While, a combination of lysine modification, tryptic digestion, and MALDI-TOF analysis unveiled that horse heart C*c* promotes the fusion of lipid vesicles via an interaction at a second positive patch. This region (*L*-site) comprises K22, K25, K27, H26, and H33, besides the previously reported *A*- and *C*-sites (Figure 2(a)) [98].

An additional UV-Vis analysis showed slight differences in the binding kinetics within a set of yeast C*c* mutants [99]. Based on their own data and the solution structure of the protein (PDB 1AKK; [100]), the authors proposed a cleft defined by the *Ω*_III_ residues K72, K73, K86, and R91, matching *A*-site. Building on from this data, the ability of site-directed mutants of horse heart C*c* to bind CL-containing liposomes was tested [101]. Notably, substituting K73 and K79 with asparagine alters the affinity of C*c* towards these liposomes, whereas the same mutation at position 72 does not.

A common feature of these binding sites is the presence of several positively charged and hydrophobic residues (Figures 2(b) and 2(c)). In line with Tollin and Salamon's early postulates [88], the interaction between the positively charged residues of C*c* and negatively charged phosphate groups of CL initiates the formation of the C*c*-CL complex driven by electrostatic interactions [101–104]. After the initiation of the complex, hydrophobic C*c* residues play a key role in the interaction with CL acyl chains, thereby establishing a tight binding between C*c* and CL [15, 105].

A strong piece of evidence supporting the extended anchorage hypothesis is the presence of a channel formed by residues 52 to 74 in the tuna C*c* XRD structure [81], where the highlighted cavities are only visible in the 4 Å model. An attempt to manually dock CL inside the structure of horse heart C*c* yielded two acyl chains within the backbone [97]. Any assessment of clashes of these two acyl chains with internal residues was missing. Nevertheless, none of the two cavities in this structure appear in the updated structure at 1.5 Å resolution (PDB 5CYT; unpublished). Hence, the extended lipid anchorage model demands that C*c* must undergo a substantial conformation change when interacting with lipid vesicles. A low-resolution analysis using monoclonal antibodies suggested that lipid-bound C*c* displayed an alkaline-like conformation [106]. The alkaline form of oxidized C*c* presents a conformation different to that of the native species, which relates to an exchange between the Met thioether ligand and a Lys amine. However, to our knowledge, the only C*c* alkaline structure available (PDB 1LMS; [107]) lacks a channel in which the acyl chain may be lodged, although the heme moiety is more accessible to solvent than the native structure. Another possibility is the formation of C*c* oligomers by domain swapping [108, 109]. Although these structures are highly variable depending on how the domain swapping is triggered by experimental conditions, a recent analysis has shown they are capable of encompassing an acyl chain [110]. Furthermore, Tyr67 in these structures would face C_11_ of docked linoleic (18 : 2^*Δ*9,12^) acid, a finding in agreement with peroxidation mechanisms. However, evidence of C*c* oligomerization in the presence of membranes remains unavailable.

**Figure 3:**
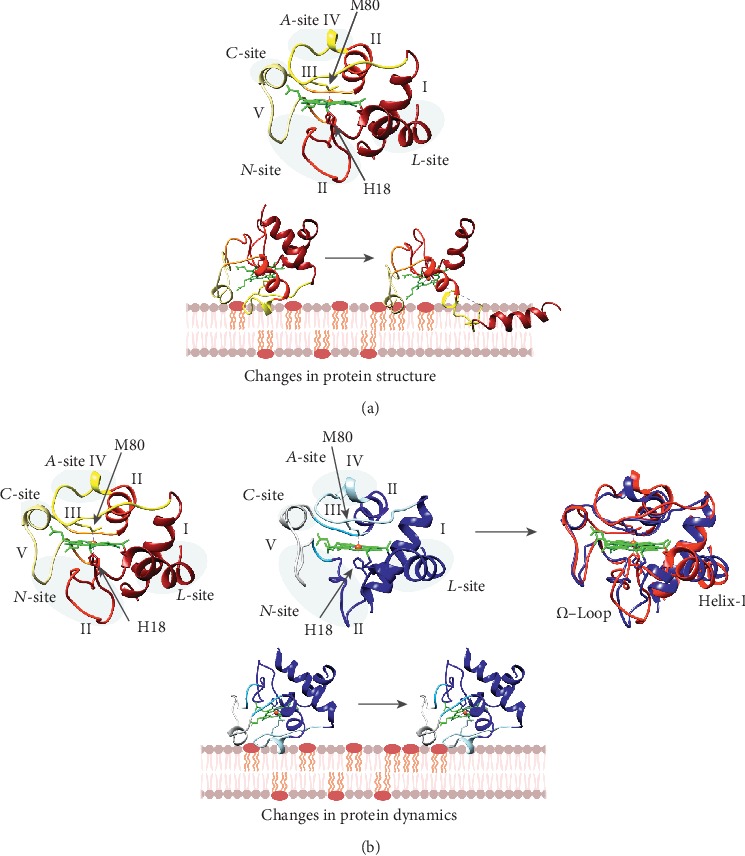
Proposed model for the interaction of cytochrome *c* with cardiolipin at pH values above 7. (a) Upper: structure of free C*c* showing the foldon units (in red scale, PDB 1AKK [100]). Lower: the C*c*-CL interaction promotes unfolding of the metalloprotein and dissociation of the axial ligand M80, thus increasing accessibility to the heme crevice. (b) Upper: structural comparison of free (in red scale, PDB 1AKK [100]) and CL-bound C*c* (in blue scale, PDB 2N3B [115]). Lower: interaction of C*c* with CL yields a slight difference in dynamics at the level of the *Ω*-loops and helix-I. The different foldon units of C*c* are colored as a gradient from the most stable (dark colors) to the weakest region (light colors). The heme group is in green, and the iron atom in orange.

Many of the studies above report the loss of the Met80 coordination and at least partial denaturation—or transition to a molten globule state—of C*c* when binding to CL-containing vesicles or liposomes [15, 84, 93, 95–97, 109–111]. Aside from CL, some other lipids can elicit similar such structural changes in C*c* [105]. This makes the heme moiety more accessible to small substrates such as carbon monoxide or nitric oxide [105, 111]. Moreover, time-resolved fluorescence resonance energy transfer (trFRET) experiments show labelled residues—not previously reported to bind CL—moving further away from the heme group in the presence of CL-containing liposomes [103, 112]. Furthermore, changes in the CD spectra and Trp59 fluorescence indicate unfolding of at least the lowest energy foldons. Consistent with the loss of Met80 coordination, substantial negative shifts in the midpoint potential of C*c* are observable in the presence of lipid vesicles [113, 114]. Disruption of the Fe-Met80 bond also correlates with a substantial increase in C*c* peroxidase activity when C*c* interacts with CL-containing vesicles [15, 105, 113]. An illustration of conformational changes, adapted from Muenzner et al. [112], is available in Figure 3(a).

The current understanding of the effects of lipid binding on C*c* structure (as described above) is controversial. For instance, depending on the nature of the CL-rich membrane preparations and experimental setup, the shift in redox potential can be positive [86] or negative [113, 114]. Recently, Wand and collaborators demonstrated that the mitochondrial crypts are concave surfaces, opposed to the convex lipoic vesicles often used in the analyses. They then analyzed the solution structure of oxidized horse heart C*c* encapsulated in reverse lipid micelles by NMR [115]. They utilized pseudocontact shifts as experimental restraints, which are highly sensitive to distances and orientation with respect to the main axes of the iron coordination sphere. An overlay of the structures of free [100] and encapsulated horse C*c* [115] (PDB 1AKK [100] and 2N3B [115], respectively) are shown in Figure 3(b). Notably, the structure of C*c* remains unaltered, and the chemical-shift perturbation map [115] resembles that observed for interactions between C*c* and its protein targets [116] and other class I single-heme cytochrome *c* family members [117]. In fact, this patch includes a novel region (*N*-site), not previously reported, comprising F36, G37, T58, W59, and K60, besides the known residues of the *A*- and *L*-sites (Figure 2(a)). Surprisingly, ssNMR spectra tracking the interaction between C*c* and small unilamellar (convex) vesicles showed no feature indicating formation of either a molten globule or unfolding [118]. Consistently, solution HSQC (NMR) spectra of horse heart C*c* in the presence and absence of similar vesicles overlapped quite well [119]. Although some signals broaden, while others were displaced—as expected for this interaction—no features of unfolding are apparent. Therefore, the overall curvature or the membrane may be irrelevant for the stability of bound C*c*. Previous data on C*c* unfolding in the presence of lipid vesicles might be reviewed in regard to membrane composition, lipid stability, and experimental setup. For instance, the dynamics of C*c* can change depending on buffer conditions [119]. Similarly, the ionization state of CL also affects C*c* binding [120].

Understanding the entire landscape of the data requires the rationale underpinning the studies to be taken into account. An equilibrium between unbounded native C*c* species and membrane-bound populations—by either weak electrostatic interactions, hydrogen bonding, or hydrophobic interactions—can be observed. The relative weights of such populations vary with experimental conditions. Furthermore, our ability to detect different species relies on the sensitivity of each biophysical approach. As recently pointed out, many of the analyses above suffer from low-resolution data or the introduction of probes that could partially alter results [118]. Therefore, full understanding requires complete knowledge of experimental conditions.

## 4. Interplay between Lipid and Cytochrome *c* Dynamics: The Compact/Extended Model

C*c* can undergo several structural transitions triggered by changes in experimental conditions such as pH [89, 90, 121–123]. For example, low-spin (*S* = 0) Fe^II^ species may turn into high-spin (*S* = 2) species in the presence of liposomes, as shown by EPR and MCD spectroscopies [89]. Notably, ionic strength and lipid-to-protein (L/P) ratio strongly influence the populations of the distinct species [15, 110, 124, 125]. These ratios relate to lipid surface coverage by C*c* molecules [125, 126]. At low L/P ratios the C*c*-coated micelles undergo coalescence—forming giant unilamellar vesicles—and precipitate. Whereas at moderate L/P ratios, C*c* promotes interactions between small unilamellar vesicles [125, 127].

The dynamic of the C*c* heme group is highly sensitive to spin state, axial ligand strength, and conformational changes. Therefore, Raman spectroscopy studies have been key in unveiling the complexity of C*c* conformation equilibria under different conditions [123, 125–128]. Hildebrandt and collaborators detected native (B1; His-Met coordination) and altered (B2) states in the presence of DOPG vesicles [125]. The B2 state comprises different species: a low-spin (B2[6cLS]), His-His-coordinated species, and two high-spin species (a pentacoordinated (B2[5cHS]) and a hexa-coordinated species (B2[6cHS])) in which a water molecule acts as the sixth ligand. The populations of the different states change according to the L/P ratio. The B2 species predominates at high L/P ratios, whereas native B1 and His-His-coordinated B2[6cLS] coexist at lower ratios. These states are also detectable by MCD [114]. The B2 bis-His-coordinated species is detectable when C*c* is absorbed onto self-assembled monolayers, with and without CL [129]. At increasing concentrations of DOPC/tetraoleoylcardiolipin (TOCL) micelles, the population of bis-His species increases, as confirmed by His-by-Asn mutations and spectroscopic analyses [130].

In addition to the L/P ratio, the content of CL and its composition influence C*c* affinity and dynamics in the bound state. Fluorescence data has indeed revealed that increasing amounts of CL favors C*c* binding to membranes [120]. The theoretical analysis therein suggests that the protonation state of CL may have a strong influence on populations of distinct membrane-bound C*c* species. However, the authors acknowledge that the formalism does not include the effects that C*c* exerts on CL distribution or membrane state (see below). Indeed, kinetic investigations have shown that the exchange rate between a native-like, compact (C) and the “extended” (E) conformations correlates with the amount of CL in the vesicles [131]. A more recent spectroscopic analysis on titration experiments by Pandiscia and Schweitzer-Stenner resulted in similar conclusions [90]. Additionally, the study highlighted that the L/P ratio also affects the relative weight of electrostatic interactions, hydrogen bonds, and hydrophobic interactions—according to ionic strength series. In summary, besides governing C*c* conformational states, the L/P ratio modulates the nature of bilayer-protein interactions. Notably, all the studies highlighted above hint at a rather peripheral binding model, with little or no embedding of C*c* into the membrane [90, 123, 125, 126, 128, 129, 131].

On the other hand, C*c* does exert a strong influence on lipid head-group dynamics, as revealed by early ^31^P ssNMR studies which demonstrated an increase in acyl chains dynamics and a restraint in the polar head groups of phospholipids [132, 133]. Interestingly, C*c* has little impact on the ^31^P ssNMR “powder” spectra of dioleoyl-phosphatidylcholine (DOPC), dioleoyl-phosphatidylethanolamine (DOPE), or DOPC/DOPE vesicles not containing CL [134]. Indeed, NMR data strongly supported CL undergoing phase separation—to form CL rafts—within mixed DOPC/CL preparations upon the addition of C*c* [132, 135]. Further, freeze-fracture electron microscopy images highlighted the ability of C*c* to promote the transition of phospholipid bilayers containing CL into non-bilayer structures, including inverted tubular (H_II_) states [133, 134]. A full isotropic signal in the ^31^P ssNMR spectra of phospholipid preparations in the presence of C*c* evinced the formation of vesicular or micellar structures when the vesicles contained CL [132, 136]. Similarly, a downfield broad signal indicated that CL mediates the formation of the H_II_ phase upon the addition of C*c*. In this sense, molecular dynamics simulations in which C*c* is in contact with a DOPC/CL membrane highlight the ability of this protein to recruit CL into rafts [118]. In addition, C*c* can induce local changes in membrane curvature when the ratio of CL increases up to 20%. Furthermore, C*c* induces pore formation in DOPC/CL giant unilamellar vesicles (GUV), as shown by confocal microscopy [137]. These pores are wide enough to allow C*c* and dextran molecules to cross the membrane.

Nevertheless, the ability of C*c* to induce membrane changes seems to be secondary regarding the activation of peroxidase activity. Addition of C*c* to large DOPC/CL unilamellar vesicles at a ca. 6 CL/C*c* ratio promotes peroxidase activity without substantially affecting ^31^P ssNMR spectra or the ^13^C frequencies of the lipid glycerol signals [138]. The major population—those accounting for less than 10% of the protein are not detectable—of C*c* in these experiments displays the same structure as the native protein *in solution*. Chemical-shift perturbation analysis revealed that residues affected include the *Ω*_III_-loop and some nearby residues (including *A*-site residues). However, changes in the dynamics of the *Ω*_III_-loop as it couples with bilayer motions are observable. This, rather than an overall unfolding, is sufficient to trigger the peroxidase activity under these conditions. In accordance with this finding, the perturbation pattern shifts when the temperature is changed or when the vesicle phospholipids are unsaturated.

The formation of C*c*-CL complex requires approximately 6 molecules of CL per C*c* molecule [15, 112, 119, 138, 139]. The values of the apparent dissociation equilibrium constant for the C*c*-CL-reduced complex are in the low micromolar range (1.4 *μ*M at pH 8.1 and 2.2 *μ*M at pH 6.5) [140], whereas in the oxidized form, they are in the high micromolar range in a two-step reaction (20 *μ*M and 42 *μ*M) [102]. Nevertheless, binding constants depend on CL composition. The affinity of C*c* towards tetra-stearyl-cardiolipin-containing vesicles is several fold higher than that for TOCL ones [102], whereas tetra-myristoyl-cardiolipin barely interacts C*c* [15]. Notably, the measured affinities correlate well with the peroxidase activity of C*c* in the presence of the respective vesicles, rather than the degree of unsaturation in the acyl chains [102].

## 5. Cardiolipin Oxidation by Cytochrome *c*

CL is particularly sensitive to auto-oxidation processes—those directly initiated by inducers, such as ROS. The proximity of its four unsaturated acyl chains allow “arm-to-arm” propagation, enhancing its reactivity [141]. Auto-oxidation takes place in several steps. A free radical (e.g., a ROS molecule) contains an unpaired electron, and this semioccupied orbital is a sink for a second electron. Polyunsaturated fatty acids (PUFA), such as linoleic or linolenic composing CL, are particularly sensitive to ROS-induced oxidation due to conjugative effects. Radicals such as superoxide, peroxyl (ROO^•^), or hydroxyl (HO^•^) sequester a hydrogen atom from the *α*-methylene carbon with respect to the first (di-) vinyl group. The resulting radical reacts immediately with O_2_ to generate a peroxyl radical. The variation of electron vacancy in the lipid radical underlies the diversity of reaction products. No matter how the lipid radical originates, it tends to propagate via a reaction with molecular oxygen, water, or other lipids. Within the process of CL signaling, there are several reactions which stand out including the addition of oxygen (to form peroxides), transfer of hydrogen atoms, addition of peroxyl radicals, and intramolecular peroxide substitution [142].

Oxidative phosphorylation and certain mitochondrial enzymes are sources of O_2_^-•^ radicals [143]. Superoxide is highly soluble in lipids but can be reduced within membranes by tocopherol and quinone and eliminated by superoxide dismutase (SOD), which transforms two superoxide molecules into a molecular oxygen and hydrogen peroxide [144]. In fact, enzymes like SOD, catalase, and peroxidases take part in active cell defense against oxidative stress [144]. H_2_O_2_ is a strong oxidant—the reduction potential for the H_2_O_2_/H_2_O pair is +1.35 V, at pH 7—but kinetically inefficient. However, the presence of iron chelates under certain pathological conditions can enhance H_2_O_2_ efficiency through Haber-Weiss-like reactions (see Equations (1)–(3)) [145]:

(1) $$\begin{array}{c} {\text{Fe}}^{3+}+{\text{H}}_{2}{\text{O}}_{2}⟶{\text{Fe}}^{2+}+{\text{O}}_{2}^{-∙}+2{\text{H}}^{+} \end{array}$$

(2) $$\begin{array}{c} {\text{Fe}}^{2+}+{\text{H}}_{2}{\text{O}}_{2}⟶{\text{Fe}}^{3+}+{\text{HO}}^{-}+{\text{HO}}^{∙}\left(\text{Fento}{\text{n}}^{\text{'}}\text{s reaction}\right) \end{array}$$

(3) $$\begin{array}{c} {\text{O}}_{2}^{-∙}+{\text{Fe}}^{3+}⟶{\text{Fe}}^{2+}+{\text{O}}_{2} \end{array}$$

The hydroxyl radical product is highly reactive, sequestering hydrogen atoms from available substrates. The resulting carbon-centered radicals may react with molecular oxygen to generate (hydro-) peroxides (Equations (4)–(6)):

(4) $$\begin{array}{c} {\text{HO}}^{∙}+\text{RH}⟶{\text{H}}_{2}\text{O}+{\text{R}}^{∙} \end{array}$$

(5) $$\begin{array}{c} {\text{R}}^{∙}+{\text{O}}_{2}⟶\text{R}{\text{O}}_{2}^{∙} \end{array}$$

(6) $$\begin{array}{c} \text{R}{\text{O}}_{2}^{∙}+{\text{H}}^{+}+{\text{Fe}}^{2+}⟶\text{ROOH}+{\text{Fe}}^{3+} \end{array}$$

Nevertheless, homeostatic cells exert a tight control over metal chelation to avoid Fenton's reactions. Indeed, a set of antioxidant agents prevent a surge in the levels of ROS. Hence, except for pathological conditions, H_2_O_2_ requires the activity of peroxidases to function efficiently as an oxidant. Within the IMM, C*c* displays both peroxidase and oxygenase activities, the latter promoting CL oxidation, while sparing other phospholipids [18, 146]. This event is crucial for the release of mitochondrial proapoptotic factors into the cytoplasm [18].

### 5.1. Peroxidase and Oxygenase Activities of Cytochrome *c* (Dr. Jeckyll and Mr. Hyde)

**Figure 4:**
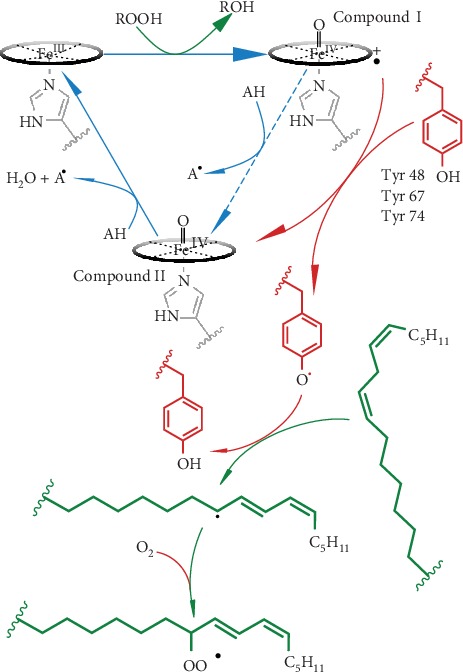
Peroxidase and oxygenase activities of cytochrome *c*. Reaction model merging the proposal from Kagan and collaborators [149] and the adapted catalytic model of cyclooxygenases as reviewed by Marnett [150]. Blue arrows correspond to the canonical peroxidase cycle [146]. Heterolytic cleavage of a peroxide substrate—preferentially for C*c*, a lipid hydroperoxide—yields the corresponding hydroxyl derivative (or water when the substrate is H_2_O_2_) and Compound I, which is reduced back to the resting ferric state in two sequential single-electron transfers from A substrate. Red and green arrows indicate the reactions purportedly leading to oxygenase activity according to the literature. Spin trap experiments have detected Y48 radicals [151]. Dimers of tyrosines 67 and 74 and oxidation products of Y48 are detectable even in the absence of H_2_O_2_ [152]. The tyrosyl radical sequesters a hydrogen atom from an unsaturated fatty acid. Finally, O_2_ reacts with the alkyl radical to form an alkyl peroxide radical as an initial product undergoing further reactions.

Heme peroxidases constitute a vital and ubiquitous group of heme enzymes catalyzing the two-electron oxidation of substrates using H_2_O_2_ as the ultimate electron acceptor [147, 148]. In canonical peroxidases, H_2_O_2_ is added to the pentacoordinated, high-spin Fe^III^. The resulting state—Compound I—is two oxidation equivalents above the resting state and is reduced back to the resting configuration in two steps through Compound II. Both states, Compounds I and II, are high valence oxoferryl (Fe^IV^) derivatives, but the first comprises an additional *π*-cation radical (see Equations (7)–(9)) (Figure 4) [147].

(7) $$\begin{array}{c} \text{Heme}{\left[\text{F}{\text{e}}^{\text{III}}\right]}_{\text{RS}}+{\text{H}}_{2}{\text{O}}_{2}⟶\text{Heme}{\left[\text{O}=\text{F}{\text{e}}^{\text{IV}}-{\text{R}}^{∙+}\right]}_{\text{CompI}}+{\text{H}}_{2}\text{O} \end{array}$$

(8) $$\begin{array}{c} \text{Heme}{\left[\text{O}=\text{F}{\text{e}}^{\text{IV}}-{\text{R}}^{∙+}\right]}_{\text{CompI}}+\text{A}{\text{H}}_{2}⟶\text{Heme}{\left[\text{O}=\text{F}{\text{e}}^{\text{IV}}\right]}_{\text{CompII}}+{\text{AH}}^{∙} \end{array}$$

(9) $$\begin{array}{c} \text{Heme}{\left[\text{O}=\text{F}{\text{e}}^{\text{IV}}\right]}_{\text{CompII}}+\text{A}{\text{H}}_{2}⟶\text{Heme}{\left[\text{F}{\text{e}}^{\text{III}}\right]}_{\text{RS}}+{{\text{AH}}^{∙}}^{∙}+{\text{H}}_{2}\text{O} \end{array}$$

In true peroxidases, a histidine residue acts as an acid-base catalyst at the distal side of the heme ring, while a highly conserved arginine residue stabilizes the alkoholate leaving group to favor the heterolytic cleavage of the peroxide O–O bond [153]. The orientation of these residues and the hydrogen-bond network at the heme distal side are critical for efficient formation of Compound I [154]. As recently pointed out by Vlasova [148], the composition of a true peroxidase active site prevents its damage by highly oxidizing intermediate compounds.

C*c* and other hemeproteins can act as pseudoperoxidases; that is, under only certain stimuli they show peroxidase activity [148, 155]. Contrary to true peroxidases, the surroundings of heme moiety are unprotected against oxidation, so the peroxidase activity ends up damaging the protein. In the early 1990s, Radi and collaborators reported the ability of C*c* to oxidize small compounds in solution [145] and to carry out lipid peroxidation [146] in the presence of H_2_O_2_. Nevertheless, the reactivity of C*c* towards H_2_O_2_ was low, as it requires the absence of the sixth ligand. Thus, the *K*_m_ value for H_2_O_2_ was very high—ca. 65 mM. In this sense, oxidative reactions showed a time lag after the addition of H_2_O_2_, indicative of an activation step. Moreover, peroxidase activity in solution decayed at pH values higher than 8 [145]. Poor reactivity may also result from the absence of histidine at the distal site (*vide infra*), as it is an acid-base residue “pumping” the heterolytic cleavage of H_2_O_2_ during Compound I formation. Based on chemiluminescence analyses of the reaction, Chance and coworkers proposed a homolytic reaction mechanism (Equations (10)–(12)) [156], supported by EPR spin trap studies of small organic hydroperoxides [157].

(10) $$\begin{array}{c} \text{Heme}\left[\text{F}{\text{e}}^{\text{III}}\right]+{\text{H}}_{2}{\text{O}}_{2}⟶\text{Heme}\left[\text{H}{\text{O}}^{∙}-\text{F}{\text{e}}^{\text{III}}\right] \end{array}$$

(11) $$\begin{array}{c} \text{H}{\text{O}}^{∙}+{\text{H}}_{2}{\text{O}}_{2}⟶{\text{H}}_{2}\text{O}+\text{H}{\text{O}}_{2}^{∙} \end{array}$$

(12) $$\begin{array}{c} \text{Heme}\left[\text{H}{\text{O}}^{∙}-\text{F}{\text{e}}^{\text{III}}\right]+\text{H}{\text{O}}_{2}^{∙}⟶\text{Heme}\left[\text{F}{\text{e}}^{\text{III}}\right]+{\text{O}}_{2}+{\text{H}}_{2}\text{O} \end{array}$$

However, EPR spin trap experiments highlighted the generation of C*c* tyrosine radicals upon treatment with H_2_O_2_ [18, 151, 155, 158]. Furthermore, spin trap experiments detecting radical products resulting from oxidation of different substrates by H_2_O_2_ strongly suggested the reaction being mediated by an oxoferryl [O=Fe^IV^] intermediate [155]. Analysis of the orientation of this radical within the native state identified a tyrosine residue at the *Ω*_II_-loop—namely, Y48 in horse heart C*c* and either Y46 or Y48 in human C*c* [159]. The peroxidase activity of C*c* in the presence of CL increases by three orders of magnitude when the driving oxidant is a lipid peroxide instead of H_2_O_2_ [149]. Spin trap analysis of reaction products by Kagan and collaborators indicates a diversity of catalytic mechanisms depending on the binding site of the substrate, namely, a homolytic peroxide cleavage minority mechanism and a major, heterolytic mechanism. Notably, the small hydroperoxide substrates involved in this pathway dock near R38 and H33. However, how the docked structure undergoes conformational changes to fulfil all geometrical constraints needed for Compound I formation remains unclear (Figure 4).

Binding of hydrogen peroxide to the heme iron is a key step in the reaction mechanism underlying peroxidase activity. The reactive species need to displace the thioether axial ligand. This takes place when a strong interaction between C*c* and a membrane induce a substantial conformational change in the hemeprotein [15, 84, 93, 95–97, 109, 111]. Nevertheless, Kagan and coworkers also detected peroxidase activity in C*c* at low CL/C*c* ratios—at which the most interactions are weak electrostatic [15]. Furthermore, they found that the energy required to activate peroxidase activity is lower than that required for partial unfolding of the protein. In fact, as pointed out before, the bond joining iron to the thioether ligand is quite weak [160]. Thus, “breathing” fluctuations of *Ω*_II_- and *Ω*_III_-loops may facilitate the replacement of the thioether ligand by small reactants—such as cyanide, carbon monoxide, water, or hydrogen peroxide—without demanding major structural changes. In fact, C*c* peroxidase activity rises in the presence of H_2_O_2_ as the concentration of denaturant increases, as previously observed in a similar analysis with bacterial cytochrome *c*_550_ [161]. Statistical analysis of activity and unfolding slopes indicate that increasing the motions of the weakest *Ω*-loops correlates well with peroxidase activity in the “compact” C*c* species [162].

The peroxidase activity of C*c* can exert a protective role in mitochondria under certain conditions [11]. Indeed, reduction of lipoid hydroperoxide compounds to hydroxyl ones provides a way of relieving oxidative stress in the mitochondrial membrane while generating signaling molecules [149]. Moreover, O_2_^-•^ reduces nitric oxide (^•^NO) generated in mitochondria under stress to form peroxynitrite (HOONO), a highly reactive species. C*c*-CL complexes have been proposed to aid peroxynitrite detoxification to yield either nitrate or nitrite through an oxoferryl state [163].

Conversely, the peroxidase activity of C*c* has been implicated in certain pathologies. For instance, oxidation of sulfite to its radical SO_3_^-•^ is a key mechanism in sulfite toxicity [164]. Moreover, C*c* mediates the formation of tyrosine radicals responsible for *α*-synuclein dimerization [66, 165], which leads to the development of the Lewy body diseases.

Canonical peroxidase activity involves two sequential one-electron oxidation steps and no transfer of oxygen from the oxoferryl complexes to the substrate [146]. Nevertheless, Compound I in certain heme enzymes—such as cytochrome P450—can transfer oxygen to certain substrates yielding hydroxy-derivatives. Interestingly, hydroxy-derivatives of CL cannot undergo peroxidation and inhibit the release of C*c* [166]. Altogether, considering the findings above concerning tyrosyl radicals in C*c* [151], Kagan and collaborators proposed that C*c* acts as an oxygenase to produce CL peroxidation [167]. This activity would also be responsible for phosphatidylserine peroxidation affecting the plasma membrane during apoptosis [65]. This hypothesis suggests that hydrogen is transferred to Compound I from a nearby tyrosine residue to yield oxoferryl Compound II and the aforementioned tyrosyl radical (Figure 4). This mechanism is similar to that proposed for cyclooxygenases, in which a hydrogen atom is sequestered from an acyl chain, generating a carbon-centered radical capable of reacting with molecular oxygen [150].

Unlike true peroxidases, the environment of the heme moiety is unprotected from highly oxidizing species arising during the catalytic cycle [148]. When reacting with H_2_O_2_, degradation of the heme porphyrin often becomes apparent by a diminution of the Soret band intensity [146, 156]. A thorough mass spectrometry analysis of C*c* residue adducts derived from H_2_O_2_ has been carried out by Flemmig and collaborators [152]. Several oxidation reactions can occur to produce a methyl-sulfoxide derivative from the methionine thioether, a sulfonic acid derivative from cysteines and 2-oxohistidine from histidine. While tyrosine residues can covalently cross-link or undergo oxidation to dihydroxyphenylalanine (DOPA) and subsequently to quinones, lysine residues can undergo carbonylation [152, 168, 169]. These changes occur when H_2_O_2_ is added to C*c* samples [80, 152, 170–173].

Remarkably, different regions in the protein display different sensitivities to oxidation depending on the environment. For instance, specific M80 oxidation takes place in the presence of DOPC/DOPE micelles [174]. The *Ω*_III_-loop is the first to be affected, whereas foldon I (helices I and IV) is the least affected by oxidation. Notably, the peroxidase activity of C*c* increases in a time-dependent manner upon the addition of H_2_O_2_. Such increments in peroxidase activity may result from successive oxidation of M80 and lysine residues, as proposed by Yin and Konermann [80, 170]. Indeed, M80 oxidation promotes conformation exchange in C*c* which impacts on heme ligation. With time, oxidative damage extends to the porphyrin ring, releasing iron capable of performing Fenton's reactions [173]. Finally, it is worth noting that CL peroxides can induce at least some of these oxidative PTM [152].

### 5.2. The Alkaline Transition of Cytochrome *c* and Peroxidase Activity

As mentioned above, previous data obtained using monoclonal antibodies highlighted that an alkaline-like conformation could interact with CL and exit mitochondria during apoptosis [106]. These antibodies also recognize the M80A mutant in the cell nucleus [175]. Notably, this mutant displays enhanced peroxidase activity. In addition, the peroxidase activity of C*c* is somewhat pH dependent [145, 176]. Indeed, for horse heart C*c*, peroxidase activity increases at acidic pH values [177] and slows beyond pH 8 [145]. Furthermore, the ability of C*c* to oxidize O_2_^-•^ falls at pH values above 7 [7]. The affinity of C*c* towards membranes and the interaction patch involved also depend on pH [15, 98, 120, 127]. These effects illustrate how pH-dependent conformation changes modulate the different activities performed by C*c*.

A number of mutations and PTM have been reported to simultaneously affect the peroxidase activity of C*c* while bringing the so-called alkaline transition to lower even physiological pH values [178–182]. Loss of M80 coordination is evident from NMR spectra and UV-Vis spectra in all these studies. C*c* peroxidase activity requires the heme iron to be pentacoordinated; the relationship with the p*K*_A_ of the alkaline transition could be attributable to the lysine amine being weaker than methionine thioether in the ligand. However, at a neutral pH, lysine is a stronger ligand than methionine [183]. In fact, horse heart and human C*c* show lower peroxidase activities at alkaline pH values [145, 182]. Moreover, mutation M100K in *P*. *versutus* cytochrome *c*_550_ makes the protein more stable at neutral pH while decreasing its peroxidase activity 20-fold [183].

Nevertheless, the shift in the alkaline transition towards lower pH values indicates destabilization or higher dynamics in the *Ω*_II_- and *Ω*_III_-loops in the Met-coordinated species. Given the weakness of the thioether ligand bond towards iron, increasing fluctuations of these loops will increase the population of high-spin species and/or alternative low-spin (e.g., bis-His) species below the p*K*_A_ value of the transition. This is observable in phosphomimic mutants, as well as in nitrated species of C*c* [178–180, 184–186]. Furthermore, enhanced dynamics facilitate the access of small substrates to the heme iron [162].

### 5.3. Control of Cardiolipin Oxidation by Post-translational Modification of Cytochrome *c*

**Figure 5:**
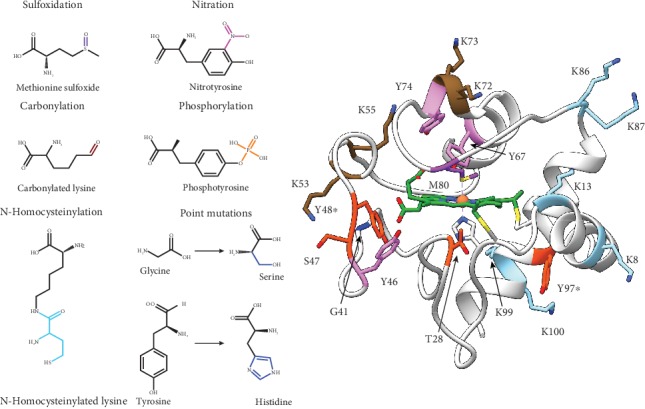
Functional implications of PTM and mutations of cytochrome *c*. Left: chemical modifications of cytochrome *c* residues. Right: ribbon representation of oxidized human C*c* (PDB 2N9J) [92]. Residues are colored by type of PTM: pink for nitration, purple for sulfoxidation, orange for phosphorylation, brown for carbonylation, cyan for N-homocysteinylation, and blue for point mutations. Y48 (asterisk) can be either phosphorylated, nitrated, or mutated for histidine, while Y97 (asterisk) can be phosphorylated or nitrated.

Protein PTM regulate tightly controlled cellular processes and increase the functional diversity of proteins, often acting as a cell response switch. Several post-translational modifications modulate C*c* structure and functionality, such as sulfoxidation [187], carbonylation [152], homocysteinylation [188], nitration [179, 180], and phosphorylation [189] (Figure 5). Phosphorylation of tyrosine residues is associated with many human pathologies including cancer, ischemia, asthma, and sepsis. As highlighted earlier, tyrosine radicals are key for the oxygenase activity of C*c* [157]. Thus, the amount of hydroxyl products from TOCL oxidation is lower when Y48E phosphomimic species instead of WT C*c* acts as a catalyst [190]. Additionally, tyrosine phosphorylation impairs the formation of radicals, preventing dimerization [191]. This fact may be critical in pathological processes such as Parkinson's disease [66]. Therefore, the PTM that affect these residues are key in regulating C*c* activity.

Given the difficulty in preserving the phosphorylation state of C*c* outside of cell extracts, a common strategy to investigate consequences of phosphorylation is to mimic the modification by site-directed mutagenesis. All phosphomimetic C*c* species, except a mutant at position 97, display altered affinity towards cardiolipin [178, 186, 190, 192–195]. The peroxidase activity of both free C*c* and C*c*-CL complexes increases in the phosphomimetic T28D and Y48*p*CMF. However, for the Y48E species, the increase only occurs with the free protein (Table 1) [178, 190, 192, 194]. In addition, at a high CL/lipid ratio, the peroxidase activity of the T28E mutant decreases [195]. A possible explanation may be that the greater population of CL versus other lipids in the liposome composition promote unfolding of the hemeprotein, acting as an off switch (Table 1) [131]. Hence, the negative charge at these positions could induce structural changes in the heme crevice which allow greater accessibility for hydrogen peroxide (Figure 5).

Peroxynitrite generated during nitrooxidative stress is a powerful amino acid modifier, affecting tyrosine residues among others [201]. Common products of the reaction between tyrosine and HOONO are 3,5-dinitrotyrosine, 3-nitrotyrosine, tyrosine radicals, and dityrosine. Nevertheless, treatment of C*c* in vitro with peroxynitrite yields its 3-nitrotyrosine adducts, with the nitro group attaching to one of the C_*ε*_ of the aromatic ring [202]. Nitration affects the redox potential of C*c* as well as its electron-exchange kinetics, depending on the residue involved [203]. The nitration of Y46, Y48, Y74, and Y97 residues also increases the peroxidase activity of C*c* and lowers the p*K*_A_ value of the alkaline transition besides other functional properties [179, 180, 183, 185, 197, 204, 205] (Table 1). Nitration of C*c* has been associated with several diseases, including chronic nephropathy [206].

All modifications/mutations of S47 and Y67 alter the peroxidase activity of C*c* [79, 179, 185, 192, 195, 197]. Y67 is located close to Met80 and is part of the hydrophobic pocket which houses the acyl chains of CL. This residue is also key for the stability of the *Ω*_III_ − loop (Table 1).

Homocysteinylation is a PTM that involves the covalent bonding of a homocysteine thiolactone—an intermediate metabolite of methionine metabolism—with a lysine residue [188]. Upregulation of this PTM is implicated in several human pathologies including cancer and cardiopathies [198, 207]. The degree of homocysteinylation, as well as the rate at which this modification occurs in the presence of homocysteine thiolactone, is related to the number of lysine residues [188]. Human C*c* displays a lysine content of 17.1% in its amino acid composition, which makes it sensitive to homocysteinylation. Homocysteinylation of surface lysines on C*c* causes aggregation of the protein. However, N-homocysteinylated lysines adjacent to the heme cavity produce conformational changes, disrupt the coordination of the M80 axial ligand, and alter the redox state of C*c*, reducing the iron of the heme group [196, 208]. These conformational changes increase C*c* peroxidase activity (Table 1) [209].

As discussed before, C*c* is modulated by several oxidative modifications due to its activity, eventually leading to changes in iron coordination. One oxidative modification is the carbonylation of lysine residues, which affects residues 72 and 73, both of which are involved in the alkaline transition of C*c* [170] (Figure 5). Reportedly, successive carbonylation events at K53, K55, K72, and K73 lead to the formation of the pentacoordinated C*c* species, resulting in increased C*c* peroxidase activity (Table 1) [170]. Similarly, the sulfoxidation of M80 facilitates the formation of a Compound I-type intermediate that initiates the activity of C*c* peroxidase (Table 1) [171, 210].

### 5.4. Peroxidase Activity of Cytochrome *c* and Diseases

Since the peroxidase activity of C*c* relates to the activation of apoptosis, it is a clear target for the development of more efficient therapies against certain diseases or pathologies. There are several examples in the literature that shed light on this topic.

The point mutations Y48H and G41S in C*c* cause the appearance of a special type of thrombocytopenia (thrombocytopenia 4) which is an asymptomatic disorder [199, 200]. Thrombocytopenia 4 is notable for normal platelet production. However, in thrombocytopenia platelets are not transported to the bone marrow and remain in the matrix, causing the effective platelet content to be lower. Both mutations cause an increase in peroxidase activity, which is related to an increase in the population of C*c* in the pentacoordinated heme state [181, 211, 212] (Table 1).

Most neurodegenerative diseases are associated with cellular stress and apoptotic processes. Due to the double role played by C*c* in the electron transport chain and apoptosis, it represents an amenable target for the development of therapies against neurodegenerative pathologies. For example, post-translational phosphorylation of C*c* at Y97 is an excellent neuroprotective strategy following brain injury as it increases the efficiency of electron transport during hypoxic conditions [193, 213]. Moreover, C*c* has been implicated in the development of Parkinson's disease—as it colocalizes with *α*-synuclein in the Lewy bodies [214]. In fact, the peroxidase activity of C*c* plays an important role in the aggregation of *α*-synuclein by tyrosine dimerization [66, 174].

Minocycline is an antibiotic functional against both Gram-positive and Gram-negative bacteria. Additionally, it displays neuroprotective properties [215]. The antibiotic minocycline impairs the interaction between C*c* and CL, inhibiting the activation of peroxidase activity and the consequent release of C*c* into the cytosol to trigger apoptosis [216–218].

Nitric oxide is a well-known inhibitor of C*c* peroxidase activity and thus may downregulate apoptosis [219, 220]. Flavonoids are excellent antioxidants that prevent cellular aging by ROS scavenger activity. In addition, they can inhibit C*c* peroxidase activity, preventing proapoptotic events [221].

The protection of healthy cells during radiotherapy is a hot topic. In fact, novel synthetic compounds—e.g., imidazole-substituted fatty acids—are currently being trialed in order to preserve healthy cells by inhibiting the peroxidase activity of C*c* during the irradiation process [222, 223]. These de novo compounds, mainly imidazole conjugates, block access to the heme crevice preventing the activation of peroxidase activity.

Finally, the activation of proapoptotic events, such as the release of C*c* after the peroxidation of CL, may be a good target for the development of more efficient and specific therapies against cancer [224, 225].

## 6. Concluding Remarks

In this review, we have outlined major advances and hypotheses regarding the oxidation of CL by C*c*. CL oxidation is a crucial event at the onset of a diverse range of pathologies, and thus, controlling it has become a key objective of current research. Targeting C*c*—a key player in CL oxidation—has emerged as an important task.

Since its discovery last century, C*c* has displayed great functional complexity despite its apparently simple structure. Indeed, its highly dynamic architecture, which enables conformation changes critical in regulating metabolism, signaling, and cell fate, still amazes the scientific community. C*c* interacts with membranes in different ways depending on their composition and curvature, being able of modifying the latter. When the hemeprotein interacts with lipids, it may undergo subtle changes in the dynamics of its most flexible foldons or may even unfold. A plethora of factors, including PTM, control these phenomena.

The chemical activity of this protein ranges from the simplest reactions to diverse and complex reaction mechanisms to drive the oxidation of substrates including CL. In the recent years, we have witnessed concerted effort to unveil the intimate chemistry of this process. Solving this conundrum will require us to discriminate minority conformations in functional assays and elucidate how this activity is tuned under distinct conditions. Nevertheless, knowledge has already amassed on the subject enabling us to examine the inhibition of CL oxidation, which will aid the development of translational approaches.

## Figures and Tables

**Table 1:** Effect of PTM and point mutations on cytochrome *c* peroxidase activity.

C*c* PTMs/mutation		Effect on peroxidase activity	References
Sulfoxidation^a^			
M80		↑	[168, 187, 196]
Nitration^b^			
Y46		↑	[184]
Y48		↑	[184]
Y67		↑	[185, 197]
Y74		↑	[185, 197]
Y97		↑	[185, 197]
Carbonylation^c^			
K53		↑	[152]
K55		↑	[152]
K72		↑	[152]
K73		↑	[152]
Phosphorylation			
T28	T28D	↑	[192]
T28	T28E	↓	[195]
S47	S47D	≈	[192]
Y48	Y48E	↑/↓	[176, 190]
	Y48pCMF	↑	[194]
Y97	Y97E	≈	[176]
	Y97*p*CMF	≈	[193]
N-Homocysteinylation^d^			
K8/K13		↑	[198]
K86/87		↑	[198]
K99		↑	[198]
K100		↑	[198]
Point mutation			
G41S		↑	[181, 199]
Y48H		↑	[181, 200]

^a^Determined under oxidative stress. ^b^Determined after peroxynitrite treatment. ^c^Determined after chloramine-T treatment. ^d^Determined after homocysteine thiolactone treatment.
